# miR-221-3p Exacerbates Obesity-Induced Insulin Resistance by Targeting SOCS1 in Adipocytes

**DOI:** 10.3390/metabo15090572

**Published:** 2025-08-27

**Authors:** Nan Li, Liang Zhang, Qiaofeng Guo, Xiaoying Yang, Changjiang Liu, Yue Zhou

**Affiliations:** 1 Center for Physical Education, Xi’an Jiaotong University, Xi’an 710049, China; xjtuchang.jiang@xjtu.edu.cn; 2 Department of Exercise Physiology, Beijing Sport University, Beijing 100084, China; kwokchiufung@bsu.edu.cn (Q.G.); zhouy@bsu.edu.cn (Y.Z.); 3 School of Strength and Conditioning Training, Beijing Sport University, Beijing 100084, China; zhangliang0102@bsu.edu.cn; 4 National Institute of Sports Medicine, General Administration of Sport of China, Beijing 100061, China; tdkfyang@163.com

**Keywords:** obesity, glucose metabolism, miRNA, PI3K/AKT pathway

## Abstract

**Objective:** Insulin resistance (IR) is a complex and multifactorial disorder that contributes to type 2 diabetes and cardiovascular disease. MicroRNAs (miRNAs) play important roles in diverse developmental and disease processes. However, the molecular mechanisms of IR are unclear. This paper aims to explore the role of miRNA in regulating IR and to elucidate the mechanisms responsible for these effects. **Methods:** IR models were created by feeding a high-fat diet (HFD) to mice or stimulating 3T3-L1 cells with palmitate. Twelve weeks of HFD trigger weight gain, leading to lipid accumulation and insulin resistance in mice. The expression profiles of miRNAs in adipose tissues (AT) from the HFD-induced mouse models were analyzed. The relationship between miR-221-3p and SOCS1 was determined using dual luciferase reporter gene assays. Metabolic alterations in AT were investigated by real-time PCR and Western blot. **Results:** miR-221-3p was significantly increased in AT. HFD-induced disturbances in glucose homeostasis were aggravated by miR-221-3p upregulation. The inhibition of miR-221-3p promoted insulin sensitivity including reduced lipid accumulation and the disruption of glucose metabolism. Of note, the 3′-UTR of SOCS1 was found to be a direct target of miR-221-3p. The SOCS1 inhibitor attenuated miR-221-3p-induced increases in IRS-1 phosphorylation, AKT phosphorylation, and GLUT4. miR-221-3p was considered to be involved in the PI3K/AKT signaling pathway, thus leading to increased insulin sensitivity and decreased IR in HFD-fed mice and 3T3-L1 adipocytes. **Conclusions:** The miR-221-3p/SOCS1 axis in AT plays a pivotal role in the regulation of glucose metabolism, providing a novel target for treating IR and diabetes.

## 1. Introduction

Obesity is a chronic metabolic disease whose incidence increases yearly. It is characterized by accumulating triglycerides (TG) in adipose tissue, which expands by adipocyte hypertrophy and hyperplasia [1]. Excessing calories stored in adipose tissues lead to metabolic abnormalities and multiple obesity-related diseases, such as chronic low-grade inflammation, dyslipidemia, hypertension, nonalcoholic fatty liver disorder (NAFLD), and cardiovascular diseases [2,3]. Insulin resistance (IR) is a key feature of obesity, metabolic syndrome, and type 2 diabetes mellitus (T2DM). The classic insulin-responsive targets are the liver, muscle, and adipose tissue (AT); the responses of these targets to insulin and other hormones determine circulating concentrations of glucose, fatty acids, and other metabolites [4,5]. Thus, it is important to understand the mechanisms of obesity-induced insulin resistance for the improvement of the treatment of diabetes.

Adipose tissue is a key organ that responds to insulin actions and, by itself, can contribute to insulin resistance. AT is not only a storage depot for excess calories but also actively secretes fatty acids, a variety of cytokines, and chemokines which could function in an endocrine or a paracrine fashion [6]. The main pathway of insulin is the phosphoinositide-3-kinase (PI3K)/protein kinase B (AKT) pathway, which regulates the metabolism of AT by promoting glucose uptake, protein synthesis, and lipogenesis [7]. Glucose transporter type 4 (GLUT4) is highly expressed in skeletal muscle and AT, which is essential in regulating systemic insulin sensitivity [8]. Ablation of GLUT4 in AT impairs systemic glucose tolerance and insulin sensitivity, the overexpression of GLUT4 can restore insulin sensitivity in mice lacking muscle GLUT4 [9,10].

MicroRNAs (miRNAs) are a class of short non-coding RNAs that function as post-transcriptional gene regulators [11]. Studies have shown that miRNAs play an important role in the development of insulin resistance in AT [1,12,13,14]. Among them, miR-221 has been extensively studied in many human malignancies and has been considered a novel diagnostic and prognostic biomarker for diseases [15]. Growing evidence shows that miR-221-3p is involved in the regulation in AT [16,17]. Furthermore, evidence indicates that miR-221-3p impairs adipocyte lipid storage and differentiation and linked with adipose tissue angiogenesis [18,19]. Suppressor of cytokine signaling 1 (SOCS1), one of the targets of miR-221-3p. miR-221-3p negatively regulates SOCS1 gene expression by binding to the 3- UTR of SOCS1 mRNAs and then suppresses the expression of the target protein SOCS1 [20]. However, the specific role of miR-221-3p in adipose tissue has not been elucidated.

While miR-221-3p has been shown to impact various cellular processes in adipocytes, the specific role of miR-221-3p in adipose tissue IR remains largely unexplored. Notably, the miR-221-3p/SOCS1 axis has not been previously investigated in the context of obesity-induced IR, making this the primary focus of our research. This study specifically aims to investigate the role and underlying molecular mechanism of miR-221-3p in adipose tissue during the development of obesity-induced IR. By analyzing the miRNA expression profile of AT in high-fat diet (HFD)-induced mice using microarray assays, we identified miR-221-3p as a key player in regulating glucose metabolism in adipose tissue. The present findings may improve and provide insight into the mechanism of obesity-induced insulin resistance and highlight miR-221-3p as a potential novel target for the treatment of diabetes.

## 2. Materials and Methods

### 2.1. Animals and Diets

C57BL/6J male mice at 4–5 weeks of age were supplied by Beijing Huafukang Laboratory Animal Technology Co., Ltd. (Beijing, China) and were maintained under the standardized conditions (22–25 °C, 12 h light/12 h dark cycle) with autoclaved water and food. The mice were randomly assigned to receive either a standard chow diet (CON; 3.87 kcal/g; 2.79% energy from fat; 1025, Huafukang) (n = 20) or a high-fat diet (HFD; 5.24 kcal/g; 60% energy from fat; H10060, Huafukang) (n = 30) for a period of 12 weeks [21]. Body weight and fasting blood glucose concentration were recorded weekly. Blood samples were obtained from the tail vein. Body fat percentage was measured by dual-energy X-ray absorptiometry (General Electric Lunar iDXA, Milwaukee, WI, USA).

### 2.2. Glycemic Control

The serum levels of fasting insulin (FINS) were measured with an ELISA kit (EZRMI-13K, Millipore, Billerica, MA, USA) using a clinical chemistry analyzer (AU5800, Beckman, Sacramento, CA, USA). For the glucose tolerance test (GTT), mice were fasted for 12 h prior to receiving an intraperitoneal injection of glucose (2 g/kg body weight). Blood samples were collected from the tail vein at 0, 30, 60, 90, and 120 min post-injection to assess glucose clearance. For the insulin tolerance test (ITT), mice were fasted for 4 h and subsequently administered insulin intraperitoneally (0.5 U/kg body weight). Blood glucose levels were measured at designated time points using a glucometer (Roche Ltd., Basel, Switzerland).

### 2.3. Histological Analysis

The epididymal fat was cross-sectioned and immediately immersed in 4% paraformaldehyde fixative solution and buffered for 48 h before histological processing. Tissues were embedded in paraffin and 5 µm sections were obtained. Subsequently, the sections were deparaffinized, dehydrated, and stained with hematoxylin and eosin (H&E) [22]. The slices stained with H&E were used to analyze adipocyte size with the Image Pro Plus 6.0 program using the 40 × image. Eight visual fields for each sample were chosen for histopathological analysis.

### 2.4. microRNA Sequencing

RNA was isolated from epididymal adipose tissue using TRIzol. Then, ligation of 3′- and 5′-adaptors was performed, and cDNA was synthesized and amplified. Library quantity was determined using the Qubit ssDNA Assay Kit (Invitrogen, Carlsbad, CA, USA), and miRNA abundance was determined by sequencing (Illumina, San Diego, CA, USA) [23]. Differential gene expression analysis was performed on TMM normalized counts with edgeR and DESeq2. miRNAs with a *p* value ≤ 0.05 and Fold Change ≥ 2.0 were considered as being significantly differentially expressed.

### 2.5. Dual-Luciferase Reporter Assay

The 3′-UTR of SOCS1, with wild-type or mutant (Mut) binding sites for miR-221-3p, was amplified and cloned into the pmiR-RB-ReportTM (RiboBio, Guangzhou, Guangdong, China) to generate the plasmid pmiR-RB-Report-m-Socs1-WT or pmiR-RB-Report-m- Socs1-MUT, respectively. For the luciferase reporter assay, 293T cells were co-transfected with the luciferase reporter vectors and miR-221-3p mimics or the corresponding negative controls (50 nM, RiboBio, Guangzhou, China) using Lipofectamine 6000 reagent. After 48 h of incubation, luciferase activity was analyzed using the Dual-Glo luciferase assay kit (50 nM, RiboBio, Guangzhou, China) according to the manufacturer’s protocol.

### 2.6. 3T3-L1 Cell Culture and Transfection

3T3-L1 pre-adipocytes (Procell Life Science Co. Ltd., Wuhan, China) were cultured in DMEM with 10% FBS, 100 U/mL penicillin, and 100 μg/mL streptomycin (Gibco, Grand Island, NY, USA) at 37 °C in a humidified atmosphere with 5% CO2. To induce differentiation, 2 days post-confluence, we used 3-Isobutyl-1- Methylxanthine (IBMX 0.5 mM), dexamethasone (250 nM), and insulin (1 μg/mL) in 10% FBS/DMEM to stimulate cells. Thereafter, the cells were further maintained in DMEM supplemented with 10% FBS and insulin [24]. The media was replaced with fresh media every 2 days until the cells were completely differentiated. The cells were used between passages 5 and 15.

After differentiation, 3T3-L1 cells were treated with 100 or 200 μM palmitic acid (PA) dissolved in 1% BSA for 24 h. Cell viability was then assessed using the CCK-8 assay (Dojindo Molecular Technologies, Tokyo, Japan) according to the manufacturer’s instructions.

Cells were seeded into a 6-well plate at a density of 1.5 × 10^5^ cells in 2 mL of culture medium per well. After seeding, 24 h later, transfections were performed using Lipofectamine 3000 (Invitrogen, Waltham, MA, USA) with miR-221-3p mimic (5′-AGCUACAUUGUCUGCUGGGUUUC-3′), miR-221-3p inhibitor (5ʹ-GAAACCCAGCAGACAAGU AGCU-3ʹ) (RiboBio, Guangzhou, China), or small interfering RNA SOCS1 (si-SOCS1, GenePharma, Shanghai, China) according to the manufacturer’s protocol. Subsequently, the cells were incubated at 37 °C for 48 h for testing.

### 2.7. Oil Red O Staining

Oil Red O staining was performed to assess lipid accumulation in cells. Briefly, 0.5 g of Oil Red O powder (O0625, Sigma-Aldrich, St. Louis, MO, USA) was dissolved in 100 mL isopropanol and subsequently diluted with distilled water in a 1.5:1 ratio. 3T3-L1 adipocytes were seeded at a density of 1 × 10^5^ cells/well, fixed with paraformaldehyde for 30 min, and then incubated with the prepared Oil Red O solution for 15 min. Following decolorization with isopropanol, the stained cells were examined using a Leica DM4 B & DM6 B microscope (Leica Microsystems, Wetzlar, Germany) at 200 × magnification. Triglyceride (TG) levels were quantified according to the manufacturer’s protocol.

### 2.8. Western Blot Analysis

Protein concentrations in cell lysates were determined using a BCA protein assay kit. A total of 30 µg of protein was separated by 10% SDS-polyacrylamide gel electrophoresis (SDS-PAGE) and transferred onto polyvinylidene fluoride (PVDF) membranes. The membranes were incubated overnight at 4 °C with primary antibodies: SOCS1 (1:500, #3950), insulin receptor substrate 1 (IRS-1) (1:1000, #2382), phosphorylated IRS-1 (p-IRS-1, Ser 302, 1:1000, #2384), PI3K (p85) (1:5000, #4292), phosphorylated PI3K (p-PI3K, Tyr 458, 1:1000, #4228), AKT (1:2000, #9272), phosphorylated AKT (p-AKT, Ser 473, 1:1000, #4060), and GLUT4 (1:1000, #2213), all sourced from Cell Signaling Technology, Inc. (Beverly, MA, USA). Glyceraldehyde-3-phosphate dehydrogenase (GAPDH, 1:5000, YM3029, Immunoway, Plano, TX, USA) was used as a loading control. After washing, the membranes were incubated with secondary antibodies at room temperature for 2 h. The anti-rabbit HRP-conjugated secondary antibody was used at a dilution of 1:10,000. Protein bands were detected using enhanced chemiluminescence, and images were captured with an Azure Biosystems C300 imaging system (Azure Biosystems, Dublin, CA, USA). Optical densities of the bands were quantified using ImageJ (RRID: SCR_003070, v 1.5.3) software.

### 2.9. Quantitative Real-Time Polymerase Chain Reaction Analyses

Total RNAs in 3T3-L1 cells were isolated using TRIzol RNA isolation reagent (Invitrogen, Carlsbad, CA, USA). Then, 500 ng RNA was reverse transcribed into cDNA using RevertAid First Strand cDNA Synthesis Kit (Thermo Fisher Scientific, Waltham, MA, USA), and the amplification was performed with 2X SG Fast qPCR Master Mix (Sangon, Shanghai, China). miRNAs were synthesized using a miDETECT A Track miRNA qRT-PCR Starter Kit (RiboBio, Guangzhou, China) according to the manufacturer’s protocol. The levels of gene expression were calculated based on the 2^−ΔΔCT^ method. The primer sequences are listed in Table 1 (all primers were synthesized by Sangon Biotech Co., Ltd., Shanghai, China).

### 2.10. Statistical Analysis

All the results are presented as the mean ± SD. Statistical analyses were performed using SPSS statistical software (v.26, SPSS Institute, Chicago, IL, USA) and GraphPad Prism software (v.10, GraphPad Software Inc., San Diego, CA, USA). Data were tested for normality using the Shapiro–Wilk test prior to statistical analysis. A repeated-measures analysis of variance (ANOVA) was used to analyze the differences in body weight, fasting blood glucose, and food intake. The two-group comparisons were conducted using unpaired Student’s t-tests. Comparisons among multiple groups were performed using one-way ANOVA followed by Tukey’s post hoc test. Correlation analyses were performed using the Pearson correlation test. Cohen’s d was used for t-tests, and η^2^ was reported for ANOVA. Effect sizes were interpreted as small (d ≈ 0.2, η^2^ ≈ 0.01), medium (d ≈ 0.5, η^2^ ≈ 0.06), or large (d ≈ 0.8, η^2^ ≈ 0.14). The number of independent experiments (N) and technical replicates (n) for each condition is stated in the figure legends. A *p* value < 0.05 was considered statistically significant.

## 3. Results

### 3.1. Mediation of Glucose Metabolism and IR in HFD-Fed Mice

**Figure 1:**
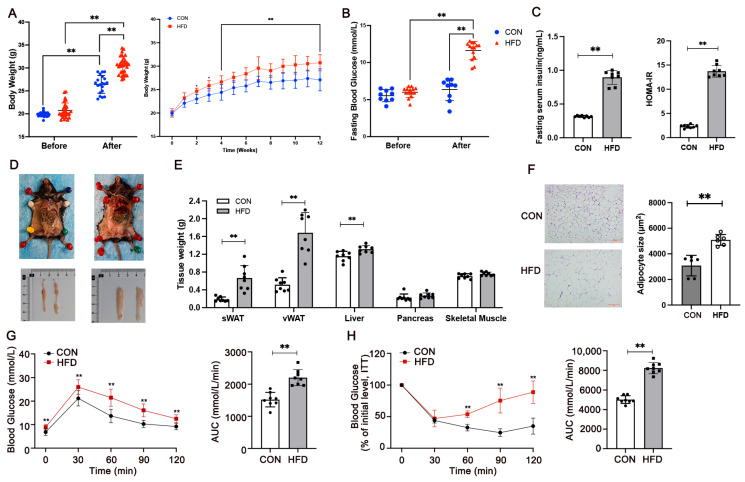
Effects of 12 weeks of high-fat diet (HFD) on body weight (**A**), fasting blood glucose (**B**), serum insulin level and the value of HOMA-IR (**C**), images of mice and epididymal adipose tissue from two groups (**D**), tissue weights (**E**), hematoxylin and eosin (H&E) staining of epididymal fat (scale bar = 100 μm), and adipocyte size (**F**). Glucose tolerance test (GTT), insulin tolerance test (ITT), and area under the curve (AUC) after 12 weeks of the high-fat diet (**G**,**H**). CON, normal control, n = 20; HFD, high-fat diet, n = 30. sWAT, subcutaneous fat, vWAT, visceral fat. Values are means ± SD (n = 8 per group). Statistical comparisons between two groups were performed using unpaired t-tests. GTT and ITT were analyzed using repeated measures analysis of variance. Body weight and blood glucose were analyzed using one-way ANOVA. ** *p* < 0.01.

After 12 weeks of dietary intervention, CON and HFD animals displayed 33% and 48% weight gain, respectively (*p* < 0.01, Figure 1A). Fasting glycemia was also significantly higher in HFD mice compared to CON mice (*p* < 0.01, Figure 1B), indicating that the high-fat diet caused impairment in glycemic control. During the 12 weeks of HFD, the increased body weight was largely attributable to the induced mass of white adipose tissue (WAT) deposits, including subcutaneous (*p* = 0.0003) and visceral fat (*p* < 0.0001) (Figure 1E). The average size of adipocytes in epididymal adipose tissues derived from HFD mice were enlarged compared with those of CON mice (Cohen’s d = 0.75, *p* < 0.01, Figure 1F). Notably, the HFD group exhibited a notably larger area under the curve (AUC) in the GTT (Cohen’s d = 0.71, *p* < 0.01, Figure 1G) and ITT than the CON group (Cohen’s d = 0.93, *p* < 0.01, Figure 1H). After 12 weeks of high-fat diet intervention, IR was diagnosed in HFD mice if their AUC during the GTT was 1.2 times higher than that of the CON group. The success rate of the IR mouse model was 73.3%. The serum insulin levels (Cohen’s d = 0.95) and HOMA-IR (Cohen’s d = 0.98) of CON mice were significantly lower than the HFD group (*p* < 0.01, Figure 1C), suggesting 12 weeks of high-fat diet feeding triggered weight gain, leading to lipid accumulation and insulin resistance in mice.

### 3.2. miR-221-3p Is Upregulated and Is Associated with Insulin Resistance in HFD-Fed Mice

**Figure 2:**
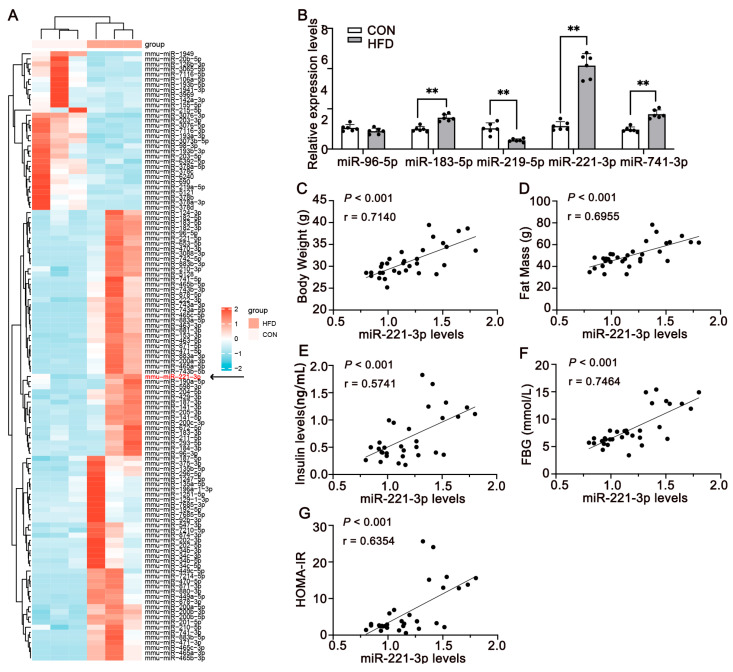
Up-regulation of miR-221-3p is associated with insulin resistance in HFD mice. (**A**) Cluster analysis of miRNA expression in AT from a normal diet (CON) or high-fat diet (HFD) for 12 weeks. Cluster in red indicates upregulation and cluster in blue indicates downregulation (N = 3). (**B**) Real-time PCR analysis of miR-221-3p expression in the AT (n = 6 per group). The association between the expression level of miR-221-3p and glucose metabolic indicators, body weight (**C**), fat mass (**D**), insulin levels (**E**), fasting blood glucose (**F**), and HOMA-IR (**G**) (n = 30). Values are means ± SD, statistical comparisons were performed using unpaired t-tests for between-CON and HFD group comparisons and correlation analysis to assess the relationship between miR-221-3p expression and metabolic parameters. ** *p* < 0.01.

To identify the factors that regulate insulin resistance in HFD mice, we performed a clustering analysis of microRNA microarrays of epididymal fat from CON and HFD mice. As a result, we found that among the differentially expressed microRNAs, miR-221-3p showed a significant upregulation in HFD mice compared to CON mice (*p* < 0.01, Figure 2A), which was further confirmed by qPCR (Table 2). Furthermore, we calculated Pearson correlations between miRNA and glucose metabolic indicators. Pearson correlation analysis showed a significant positive correlation between the expression of body weight (*p* < 0.001, r = 0.71), fat mass (*p* < 0.001, r = 0.7140), insulin levels (*p* < 0.001, r = 0.70), fasting blood glucose (*p* < 0.001, r = 0.57) and HOMA-IR (*p* < 0.001, r = 0.64), and the expression of miR-221-3p in the HFD mice (Figure 2C–G). These results suggest that miR-221-3p is highly involved in adipose tissue and is associated with insulin resistance in HFD-fed mice.

### 3.3. miR-221-3p Exacerbates Lipid Accumulation in 3T3-L1 Cells

**Figure 3:**
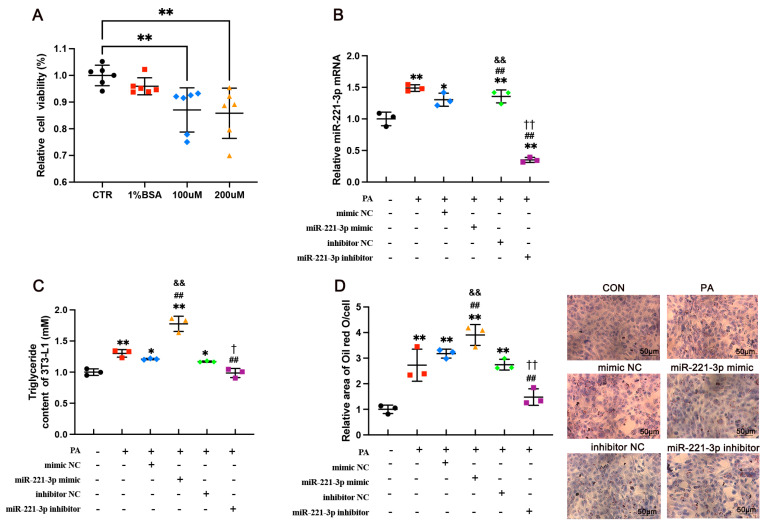
Transfection of miR-221-3p into cultured 3T3-L1 cells exacerbates lipid accumulation in vitro. (**A**) CCK-8 assay detection of 3T3-L1 cell viability after treatment with 100 or 200 mmol/L palmitate (PA) for 24 h. (**B**) Real-time PCR analysis of miR-221-3p expression in the cells treated with miR-221-3p mimic or inhibitor. (**C**) Triglyceride levels and (**D**) Oil Red O staining in cultured 3T3-L1 cells (scale bar  =  50 µm). Values are means ± SD, N = 3 independent experiments, n = 6 technical replicates per experiment. Statistical comparisons were performed using one-way ANOVA followed by Tukey’s post hoc test. * *p* < 0.05, ** *p* < 0.01 vs. control group. ^##^ *p* < 0.01 vs. PA group. ^&&^ *p* < 0.01 vs. mimic NC group. ^†^ *p* < 0.05, ^††^ *p* < 0.01 vs. inhibitor NC group.

We then reproduced the adipose tissue microenvironment with PA to elucidate the effects of miR-221-3p in vitro on lipid accumulation. The cells were cultured with different concentrations of PA for 24 h. Cell proliferation was inhibited in the PA treatment at a dose of 0.1 mM (η^2^ = 0.15, *p* = 0.0092) and 0.2 mM (η^2^ = 0.16, *p* = 0.0045, Figure 3A), and there was not a major difference between the two concentrations. Therefore, we used a 100 μmol/L concentration of PA in subsequent experiments. The cultured 3T3-L1 cells were transfected with a miR-221-3p mimic and inhibitor. RT-PCR was used to detect the level of miR-221-3p in each group (Figure 3B). miR-221-3p levels of PA (η^2^ = 0.81, *p* = 0.0051), mimic NC (η^2^ = 0.72, *p* = 0.0825), miR-221-3p mimic (η^2^ = 0.88, *p* < 0.0001), and inhibitor NC (η^2^ = 0.04, *p* = 0.0375) were significantly higher than in the control group, and miR-221-3p inhibitor levels were significantly lower than in the control group (η^2^ = 0.85, *p* = 0.0005). The miR-221-3p inhibitor reduced lipid accumulation in 3T3-L1 cells, as evidenced by TG levels (Figure 3C). In contrast, miR-221-3p mimic significantly increased TG levels (Cohen’s d = 7.95, *p* = 0.0014), while miR-221-3p inhibitor significantly decreased TG levels compared to inhibitor NC (Cohen’s d = −4.31, *p* = 0.0125). In addition, the levels of Oil Red O staining demonstrated that the miR-221-3p inhibitor significantly reduced lipid accumulation compared to the inhibitor NC (Cohen’s d = −5.69, *p* = 0.0047). The comparison between miR-221-3p mimic and mimic NC revealed an effect size of Cohen’s d = 2.85 (*p* = 0.0465), also indicating a large effect (Figure 3D). These results show that miR-221-3p expression is up-regulated by high palmitic acid, and transfection of miR-221-3p increased lipid droplet accumulation in 3T3-L1 adipocytes.

### 3.4. miR-221-3p Expression Is Associated with GLUT4 Expression and PI3K/AKT Axis Activation in IR Cells

**Figure 4:**
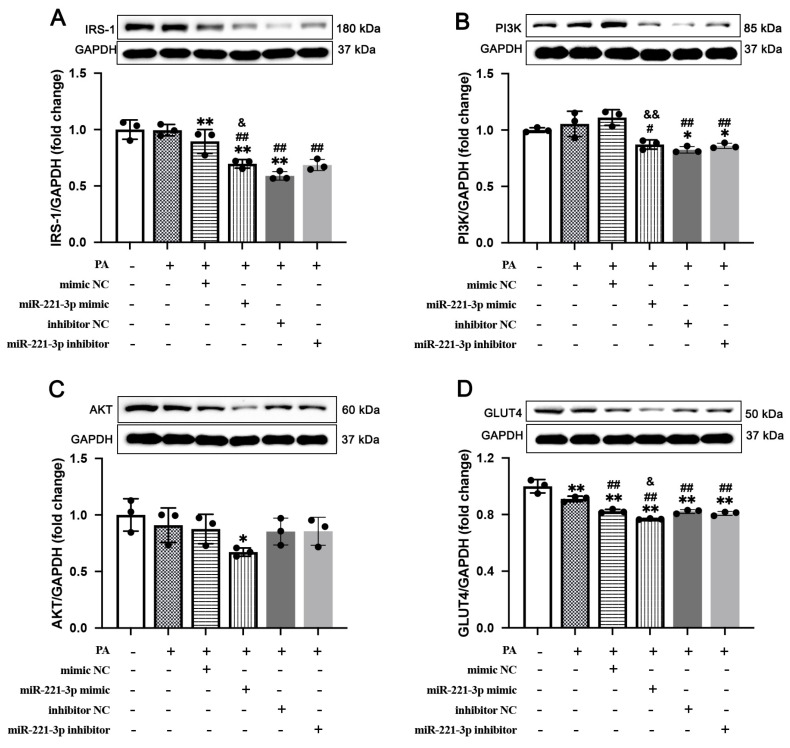
Transfection of miR-221-3p into cultured 3T3-L1 cells exacerbates insulin resistance in vitro. Representative Western blotting and summarized data of IRS-1 (**A**), PI3K (**B**), AKT (**C**), and GLUT4 (**D**) in the 3T3-L1 cells (N = 3 independent experiments, n = 3 technical replicates per experiment). Statistical comparisons were performed using one-way ANOVA followed by Tukey’s post hoc test. Values are means ± SD, * *p* < 0.05, ** *p* < 0.01 vs. control group. ^#^ *p* < 0.05, ^##^ *p* < 0.01 vs. PA group. ^&^ *p* < 0.05, ^&&^ *p* < 0.01 vs. mimic NC group.

As shown in Figure 4, the treatment of PA and miR-221-3p significantly induced the expression of insulin resistance-related proteins. We found PA could significantly down-regulated the expression of insulin resistance-related proteins. Moreover, miR-221-3p mimics significantly exacerbated the expression of IRS-1 (Cohen’s d = −3.07, *p* = 0.0374, Figure 4A), PI3K (Cohen’s d = −5.12, *p* = 0.0069, Figure 4B), and GLUT4 (Cohen’s d = −6.85, *p* = 0.0024, Figure 4D). Consistently, treatment with miR-221-3p inhibitors reversed these changes in PA treatment cells (*p* < 0.01). Therefore, these results suggest that miR-221-3p plays an important role in insulin resistance and exacerbates disturbances in glucose homeostasis.

### 3.5. miR-221-3p Targets Socs1 and Regulates Its Expression

**Figure 5:**
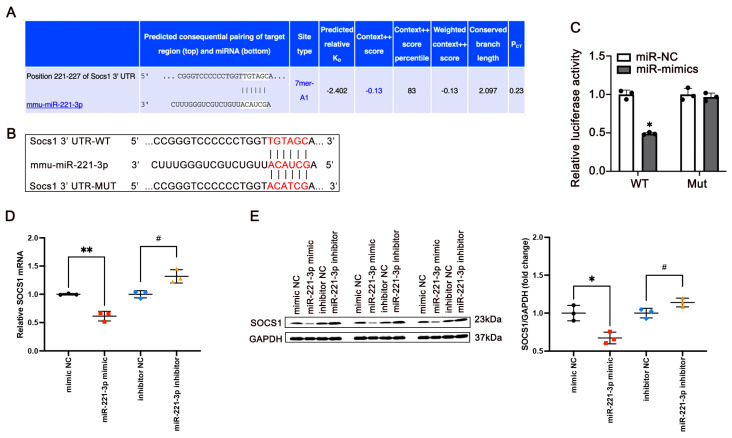
MiR-221-3p specifically targets the suppressor of cytokine signaling 1 (*Socs1*) and regulates its expression. (**A**) The binding sequence of miR-221-3p for SOCS1 was retrieved by the TargetScan database. (**B**,**C**) Dual-luciferase reporter assay identified the target sites between miR-221-3p and SOCS1 3′UTR (N = 3 independent experiments, n = 3 technical replicates per experiment). The effects of overexpression and silencing of miR-221-3p on SOCS1 mRNA level were detected by qRT-PCR (**D**) and Western blot (**E**). Values are means ± SD, statistical comparisons were performed using one-way ANOVA followed by Tukey’s post hoc test. * *p* < 0.05, ** *p* < 0.01 vs. mimic NC group. ^#^ *p* < 0.05 vs. inhibitor NC group.

Next, we investigated the potential mechanisms by which miR-221-3p is regulated in IR. The TargetScan database predicted and showed the binding sequence of miR-221-3p for the 3′UTR of Socs1 (Figure 5A). To determine whether miR-221-3p binds to the 3′UTR of Socs1, we conducted a luciferase assay (Figure 5B). The results showed that the luciferase activity of cells co-transfected with Socs1-WT and miR-221-3p mimic was significantly reduced (Figure 5C, *p* < 0.05), and no significant changes were observed in the mutated Socs1 region, suggesting that miR-221-3p binds to the 3′UTR of Socs1. In addition, overexpression of miR-221-3p suppressed the mRNA level of Socs1 (Cohen’s d = −7.7, *p* = 0.0015) as well as its protein expression (Cohen’s d = −2.81, *p* = 0.0480, Figure 5D), while miR-221-3p inhibitor produced a reverse regulatory effect (Cohen’s d = 4.07, *p* = 0.0152 for mRNA level of Socs1, Cohen’s d = 2.82, *p* = 0.0152 for protein expression, Figure 5E). According to these results, miR-221-3p can directly regulate the expression of Socs1 through these binding sites.

### 3.6. miR-221-3p Inhibition Protects Against Glucose Metabolism and Insulin Resistance Induced by PA In Vitro by Binding to SOCS1

**Figure 6:**
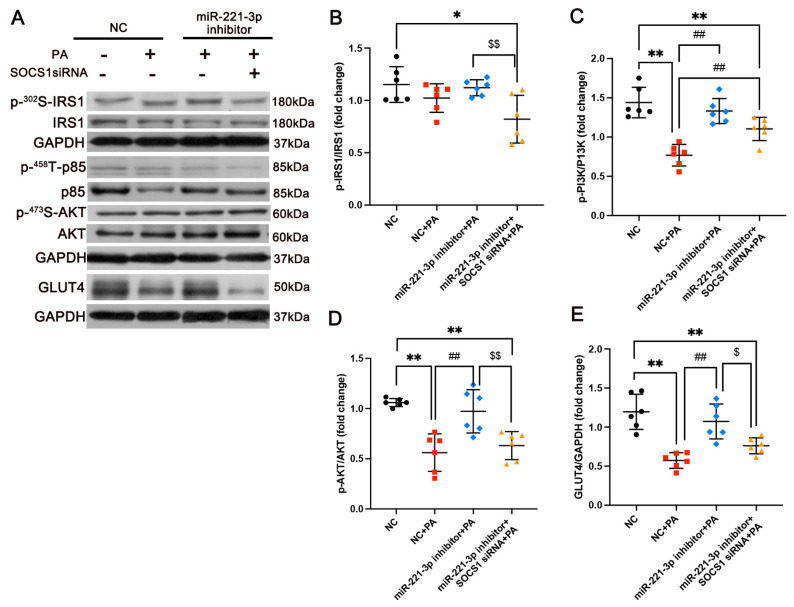
miR-221-3p inhibition protects glucose metabolism and insulin resistance in 3T3-L1 by binding to SOCS1. (**A**) Western blotting (WB) analysis of proteins. Representative WB of p-IRS1/IRS1 (**B**), p-PI3K/PI3K (**C**), p-AKT/AKT (**D**), and GLUT4 (**E**) in 3T3-L1 cells (N = 3 independent experiments, n = 6 technical replicates per experiment). Values are means ± SD, statistical comparisons were performed using one-way ANOVA followed by Tukey’s post hoc test. * *p* < 0.05, ** *p* < 0.01 vs. control group. ^##^ *p* < 0.01 vs. PA group, inhibitor NC group. ^$^ *p* < 0.05, ^$$^ *p* < 0.01 vs. miR-221-3p inhibitor group.

To further confirm the role of miR-221-3p/SOCS1 in glucose metabolism and IR in 3T3-L1, cells were transfected with miR-221-3p inhibitor or SOCS1 siRNA. The siRNA-induced SOCS1 knockdown blocked the protective effects of miR-221-3p inhibitor on glucose metabolism and IR after the PA treatment (Figure 6A). It was found that the PA treatment induced decreased expression levels of phosphorylated PI3K (η^2^ = 0.84, *p* < 0.01, Figure 6C) and phosphorylated AKT (η^2^ = 0.745, *p* < 0.01, Figure 6D), and the levels of GLUT4 (η^2^ = 0.16, *p* < 0.01, Figure 6E), all of which were blocked by miR-221-3p inhibition (*p* < 0.01). siRNA-SOCS1 partially reversed the regulatory impact of miR-221-3p inhibitor on phosphorylated IRS-1 (η^2^ = 0.507, *p* < 0.01, Figure 6B), phosphorylated AKT (η^2^ = 0.577, *p* < 0.01, Figure 6D), and GLUT4 (η^2^ = 0.490, *p* < 0.05, Figure 6E). These findings suggest that miR-221-3p inhibition reduces glucose intolerance and insulin resistance in 3T3-L1 by binding to SOCS1.

## 4. Discussion

Dysregulation of adipose tissue engenders various complications, including ectopic lipid accumulation and systemic insulin resistance and contributes to the development of obesity and T2DM, but the effectors are incompletely understood. In this study, we found that miR-221-3p was significantly upregulated in the AT of HFD mice and promoted metabolic dysregulation and insulin resistance. miR-221-3p inhibition could attenuate PA-induced lipid abnormalities and glucose metabolism. More importantly, we proposed that the miR-221-3p/SOCS1 axis may play a crucial role in this pathological process. Our findings may provide new insights into the pathogenic mechanisms of obesity and T2DM.

Diet-induced obesity is linked with insulin resistance and results from an imbalance between energy intake and expenditure, leading to excessive accumulation and storage of adipose tissue. Long-term dysregulation of lipid accumulation and adipose dysfunction could often result in disease states, including metabolic dysfunction, chronic inflammation, and T2DM [25]. Increased fat and caloric intake were regarded as the main cause of adiposity. When mice were fed a high-fat diet with 60% of total energy as fat, it led to excess energy intake and eventual obesity [26,27]. In our study, we found that mice fed with an HFD (60% fat content) exhibited induced weight gain, body fat content, insulin levels, and significantly accumulated adiposity. Moreover, obesity and elevated serum insulin levels have been shown to be associated with insulin resistance [28]. As noted, a high-fat diet causes hyperinsulinemia, insulin resistance, and perhaps other maladies. Insulin as a major regulator plays an important role in the regulation of glucose and lipid metabolism [29,30,31]. The excessive accumulation of free fatty acids in the blood promotes hepatic glucose production to raise blood glucose levels, which aggravates insulin resistance. Elevated blood concentrations of fatty acids induce inflammatory cytokine release, block insulin receptor signaling through inhibition of insulin receptor substrates (IRS), and provoke insulin resistance [32]. In the present study, we found that after 12 weeks of HFD, mice displayed high levels of fasting blood glucose and serum insulin, which led to impaired glucose tolerance and insulin tolerance.

MicroRNAs (miRNAs) are small non-coding RNAs that post-transcriptionally regulate gene expression [33]. miRNAs have been revealed to play important roles in T2DM and its related complications. Dysregulation of miRNAs from insulin-sensitive organs or tissues have been implicated in the pathogenesis of diabetes and insulin resistance [34]. Previous studies have found that miR-802 is increased in the pancreatic islets of mouse models with obesity and overexpression of miR-802 in mice causes impaired insulin transcription and secretion [35]. Moreover, it was found that adipose cell-derived miR-27a induced the activation of macrophages and insulin resistance in high-fat-diet-fed mice through inhibition of PPAR-γ [36]. In comparison, miR-221-3p also regulates insulin resistance [37]. Our microarray analysis showed that miR-221-3p is significantly upregulated in HFD mice and positively correlates with glucose and lipid metabolism. Similarly to miR-802 and miR-27a, miR-221-3p contributes to insulin resistance in adipose tissue. Ahonen, Asghar [19] found that miR-221-3p overexpression inhibited terminal differentiation of human adipocytes, whereas the miR-221-3p inhibitor increased TG storage. The overexpression of miR-221-3p is linked to enhanced adipogenesis. Elevated miR-221-3p impairs adipocyte lipid storage and differentiation, which are relevant for metabolic diseases and affect cancer progression [38]. In our study, we mimicked the adipose tissue microenvironment with palmitate and investigated whether levels of miR-221-3p significantly increased in 3T3-L1 cells. Overexpression of miR-221-3p increased triglyceride accumulation and inhibited glucose uptake levels. miR-221-3p inhibition could effectively decrease triglyceride levels and increase glucose uptake in fat. Lifestyle interventions such as regular physical exercise have been shown to effectively suppress miR-221-3p expression, which can potentially mitigate the progression of obesity-induced insulin resistance and type 2 diabetes, ultimately improving insulin sensitivity [39,40]. Therefore, our data indicate that miR-221-3p is important in glucose metabolism but the regulations by miR-221-3p in insulin resistance deserve further investigation.

Based on bioinformatics analysis, SOCS1 was confirmed as a target of miR-221-3p. Our study suggests that miR-221-3p led to the development of IR in adipocytes and mice with obesity, at least partially, by targeting the 3′-UTR of SOCS1. Consistent with our findings, Navarro, Pairet [41] also identified a targeting relationship between miR-221-3p and SOCS1 in patients with thrombocytosis. SOCS1 has been reported as a key cytokine in the immune response [42]. Inhibition of miR-221-3p has been shown to increase the expression of SOCS1, reduce levels of NF-κB and IL-6, and consequently decrease inflammation levels [43]. As the most important member of the Suppressor of Cytokine Signaling (SOCS) family, SOCS1 also has key roles in regulating insulin signaling in different tissues [44]. SOCS1 is able to interact with insulin receptors in cells, with consequent effects on IRS1 phosphorylation [45]. Although previous studies have shown that SOCS1 inhibits IRS1 phosphorylation, leading to impaired insulin signaling [46,47], we observed that it promotes IRS1 expression in our study. This finding may suggest that the regulation of insulin signaling is context-dependent and tissue-specific. The PI3K/AKT signaling pathway is a critical pathway that plays a key role in glucose metabolism [48]. Excessive high-fat diet interferes with the insulin signaling pathway in adipose tissues, which leads to insulin resistance. Insulin acts via the IR and IRS-1 to phosphorylate and thereby activate AKT. Activated AKT stimulates the translocation of glucose transporter GLUT4 to the plasma membrane, and facilitates glucose uptake and its transfer into adipocytes [49,50]. Li, Song [51] found that miR-221-3p induced IR by inhibiting levels of IRS-1 and phosphorylated AKT in the liver and skeletal muscle. Notably, we confirmed that glucose uptake was increased due to miR-221-3p downregulation in PA-induced 3T3-L1 cells. In addition, the siRNA-induced SOCS1 blocked the effects of miR-221-3p inhibitor, which contributed to an increase in glucose and insulin sensitivity in adipocytes. Our study revealed that siRNA SOCS1 reversed the inhibitory effects of miR-221-3p inhibition on PA-induced insulin resistance in 3T3-L1 cells. These results indicate that miR-221-3p may exert its role through the PI3K/AKT pathway.

## 5. Limitation

Nevertheless, there are certain shortcomings in the current study. Insulin resistance is a complex pathological condition; beyond miR-221-3p, many other miRNAs show significant regulation abilities in HFD mice. Additionally, many key genes of glucose metabolism have proved to be targets of miR-221-3p. In the future, we would also explore more functions between miRNA and insulin resistance. Through potential delivery systems, the effectiveness of in vivo delivery of miR-221-3p inhibitors could be explored, with clinical trials necessary to assess their safety and efficacy in improving insulin sensitivity. This approach could offer novel therapeutic strategies for obesity-related insulin resistance and type 2 diabetes.

## 6. Conclusions

**Figure 7:**
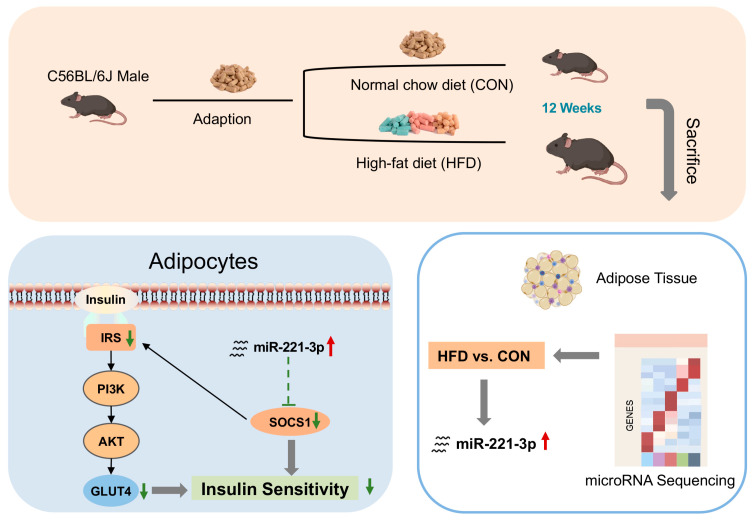
miR-221-3p exacerbates obesity-induced insulin resistance by targeting SOCS1 in adipocytes.

In summary, the present study reported that the up-regulation of miR-221-3p expression significantly affected insulin sensitivity and glucose metabolism in high-fat diet mice and adipocytes. MiR-221-3p plays a considerable role in promoting IR, at least in part through the SOCS1-mediated PI3K/Akt signaling pathway (Figure 7). Our study suggests that miR-221-3p might be a new target for the treatment of improving IR during the progression of T2DM. Further clinical studies and trials are needed to evaluate the safety and efficacy of miR-221-3p inhibitors as a therapeutic strategy for metabolic diseases. Meanwhile, other miRNAs and their interactions with insulin resistance should be investigated to provide a more comprehensive understanding of the mechanisms involved.

## Figures and Tables

**Table 1:** Primer sequences.

Gene	Primer Sequences (5′-3′)	
*Socs1*	F	CCAACGGAACTGCTTCTTCG
	R	AAAGGCAGTCGAAGGTCTCG
*β-actin*	F	GTGCTATGTTGCTCTAGACTTCG
	R	ATGCCACAGGATTCCATACC
miR-221-3p	RT	CTCAACTGGTGTCGTGGAGTCGGCAATTCAGTTGAGGAAACCCA
	F	ACACTCCAGCTGGG AGCTACATTGTCTGC
miR-183-5p	RT	CTCAACTGGTGTCGTGGAGTCGGCAATTCAGTTGAGAGTGAATT
	F	ACACTCCAGCTGGGTATGGCACTGGTAG
miR-219a-5p	RT	CTCAACTGGTGTCGTGGAGTCGGCAATTCAGTTGAG AGAATTG
	F	CACTCCAGCTGGG TGATTGTCCAAACG
miR-741-3p	RT	CTCAACTGGTGTCGTGGAGTCGGCAATTCAGTTGAG TCTACATA
	F	ACACTCCAGCTGGG TGAGAGATGCCATTC
miR-96-5p	RT	CTCAACTGGTGTCGTGGAGTCGGCAATTCAGTTGAGAGCAAAAAT
	F	ACACTCCAGCTGGG TTTGGCACTAGCAC
All R		TGGTGTCGTGGAGTCG
U6	F	CTCGCTTCGGCAGCACA
	R	AACGCTTCACGAATTTGCGT

**Table 2:** Key miRNAs in Adipose Tissue.

miRNA	Log FC	HS-NS
mmu-miR-183-5p	1.13	up
mmu-miR-219a-5p	−1.50	down
mmu-miR-221-3p	3.40	up
mmu-miR-741-3p	2.84	up
mmu-miR-96-5p	2.34	up

## Data Availability

The original contributions presented in this study are included in the article/Supplementary Materials. Further inquiries can be directed to the corresponding author.

## Supplementary Materials

The following supporting information can be downloaded at: https://www.mdpi.com/article/10.3390/metabo15090572/s1, Table S1: Antibody information; Table S2: Original data of Western blotting.
